# Robust Interacting Multiple Model Filter Based on Student’s *t*-Distribution for Heavy-Tailed Measurement Noises

**DOI:** 10.3390/s19224830

**Published:** 2019-11-06

**Authors:** Dong Li, Jie Sun

**Affiliations:** 1 Unit 94, PLA 91550, Dalian 116023, China; 2 Unit 93, PLA 91550, Dalian 116023, China; sunshine_scholar@163.com

**Keywords:** IMM, filter, robust, heavy-tailed measurement noises, Student’s *t*-distribution, variational Bayesian

## Abstract

In maneuvering target tracking applications, the performance of the traditional interacting multiple model (IMM) filter deteriorates seriously under heavy-tailed measurement noises which are induced by outliers. A robust IMM filter utilizing Student’s *t*-distribution is proposed to handle the heavy-tailed measurement noises in this paper. The measurement noises are treated as Student’s *t*-distribution, whose degrees of freedom (dof) and scale matrix are assumed to be governed by gamma and inverse Wishart distributions, respectively. The mixing distributions of the target state, dof, and scale matrix are achieved through the interacting strategy of IMM filter. These mixing distributions are used for the initialization of time prediction. The posterior distributions of the target state, dof, and scale matrix conditioned on each mode are obtained by employing variational Bayesian approach. Then, the target state, dof, and scale matrix parameters are jointly estimated. A variational method is also given to estimate the mode probability. The unscented transform is utilized to solve the nonlinear estimation problem. Simulation results show that the proposed filter improves the estimation accuracy of target state and mode probability over existing filters under heavy-tailed measurement noises.

## 1. Introduction

The Kalman filter is widely used for target tracking due to the low computational complexity for real time processing and the statistic optimality under a linear state space model with Gaussian noises. Because of the model uncertainty of target maneuvering and the heavy-tailed measurement noises deduced by outliers, the performance of Kalman filter may break down dramatically, and even the convergence cannot be guaranteed. Addressing model uncertainty and investigating robust filter against heavy-tailed measurement noises are two important research topics in the target tracking field.

Model uncertainty in maneuvering target tracking exhibits that the motion model of target is time varying and that the switching time between different models is unknown. Due to the model uncertainty, the model used by Kalman filter may not match the actual dynamics so that the filter may gradually diverge. Multiple-model (MM) methods are considered as the mainstream approach to maneuvering target tracking under model uncertainty [1]. These methods establish a finite set of models to describe the different motion patterns for maneuvering target, and achieve the ultimate estimate of state by a certain combination of the estimates from each model. In the literature, the MM methods can be categorized into two different types: fixed-structure MM (FSMM) methods which use a fixed model set at all times and variable-structure MM (VSMM) methods in which the model set is not fixed. The generalized pseudo-Bayesian estimator of order *n* (GPBn) filter and the interacting multiple model (IMM) filter [2] are the typical FSMM methods. Among them, the IMM filter is considered to be the best compromise between complexity and performance, and has been successfully applied in a large number of tracking applications [3,4,5,6,7,8]. In IMM filter, several sub-filters operate in parallel and cooperate with each other through an interacting strategy, leading to improved performance of estimation. For the FSMM methods, if the model set used does not match the set of actual models of target movement, the performance can be degraded. To cope with this problem, several VSMM methods have been proposed, such as likely mode set (LMS) filter [9], expected-mode augmentation (EMA) filter [10], equivalent-model augmentation (EqMA) filter [11], and hybrid grid multiple model (HGMM) filter [12]. These filters use different model-set adaptation schemes to adjust the model set. For example, the EqMA filter augments the model set by a new variable model depending directly on the history information from the recent models, while the HGMM filter adapts the model set by the hybrid grid scheme which consists of a fixed coarse grid and an adaptive fine one. In recent years, some researchers have proposed Sequential Monte Carlo (SMC) methods [13,14] for dealing with the problem of model uncertainty in target tracking. These methods maintain several particle filters (PFs) in parallel and fuse the results of PFs to provide the posterior probability of model and the posterior distribution of state. In addition, the SMC method in [14] uses a scale parameter to detect the model change and does not require the mode transition probabilities. This strategy of handling model change is quite different from the above mentioned FSMM and VSMM methods.

Besides the model uncertainty, the outlier measurements existing in real tracking scenarios should also be handled. Outliers can induce heavy-tailed non-Gaussian noises, and the performance of Kalman filter may be seriously degraded since its underlying optimality criterion is minimum mean square error, which is very sensitive to heavy-tailed noises. A large number of robust filters have been proposed to improve the robustness against non-Gaussian noises. The Huber-based filter [15,16,17,18,19] is a classical robust method based on a combined minimum *l*_1_ and *l*_2_ norm estimation. The maximum correntropy Kalman filter (MCKF) [20,21,22,23,24] is another effective way to suppress the impact of non-Gaussian noises. It uses the robust maximum correntropy criterion (MCC) as the optimality criterion. Simulation experiments show that the MCKF outperforms Huber-based filter in estimation accuracy [20,22,23,24]. However, neither Huber-based filter nor MCKF takes advantage of the inherent heavy-tailed feature of the noise distribution. Thus, their estimation precision is limited. The Student’s *t*-distribution is robust to outliers since it has heavier tail compared with Gaussian distribution. Based on this idea, many robust filters utilizing Student’s *t*-distribution has been proposed [25,26,27,28,29,30]. The Student’s *t* filter [25,26] is derived by modeling the posterior distribution of the state, the heavy-tailed process and measurement noises as Student’s *t*-distributions. The filter recursions are designed according to the framework of Bayesian filter. This filter may induce significant bias since an approximated method of moment matching is employed to prevent the growth of the degrees of freedom (dof) of Student’s *t*-distribution. The variational Bayesian (VB) based Student’s *t* filter [27,28,29,30] represents the Student’s *t*-distribution of noises as an infinite mixture of Gaussians, and employs VB approach to jointly estimate the state and the unknown parameters of the Student’s *t*-distribution. This filter can achieve higher estimation accuracy than the Student’s *t* filter [29,30].

In maneuvering target tracking, the problems of model uncertainty and heavy-tailed measurement noises could exist simultaneously. Investigating robust IMM filter to cope with both model uncertainty and heavy-tailed noises is meaningful. As far as we know, Shen et al. [31] considered utilizing Student’s *t*-distribution to improve the robustness of IMM filter for the first time. They modeled the heavy-tailed measurement noises as Student’s *t*-distribution and employed IMM and VB approaches to estimate the target state, the probability of motion mode and the parameters of noises. However, the dof of Student’s *t*-distribution, which is determined by the estimated parameters of an auxiliary random variable, becomes very large after several filtering steps. Then, the Student’s *t*-distribution converges to Gaussian distribution and loses the heavy-tailed property. Therefore, the robustness of this filter against heavy-tailed measurement noises is limited. In addition, the scale matrix of Student’s *t*-distribution in this filter is assumed to be known exactly. However, this premise is usually unavailable since the scale matrix is unknown or time varying in many actual applications. Furthermore, this filter is based on linear systems and should be generalized to nonlinear systems which are more common in maneuvering target tracking.

Motivated by the above discussions, we propose a new robust IMM filter to better handle the heavy-tailed measurement noises and the system nonlinearity. The posterior distribution of target state is approximated by Gaussian distribution, and the measurement noises are modeled as Student’s *t*-distribution. The unknown dof and scale matrix of the Student’s *t*-distribution are assumed to obey gamma and inverse Wishart distributions, respectively. Then, the state and the parameters of gamma and inverse Wishart distributions for each mode are interacted via IMM mixing, and they are jointly estimated by using VB approach. An approximation method is given to derive the mode-conditioned predicted likelihood which is used for calculating the mode probability. The unscented transform (UT) is employed to tackle the system nonlinearity. The growth of dof estimates is prevented and the estimation accuracy of state is improved over existing filters under heavy-tailed measurement noises as shown in our simulation example.

## 2. System Model and Assumptions

Consider the following jump Markov system

$$\left\{ \begin{array}{l} x_{k}=F_{k-1}(x_{k-1},r_{k})+w_{k-1}(r_{k})\\ z_{k}=H_{k}(x_{k},r_{k})+\varepsilon_{k} \end{array}\right.$$

where k is the scan time index, $x_{k}\in R^{n}$ and $z_{k}\in R^{m}$ are the state and measurement vectors respectively, $r_{k}$ is the mode of target movement taking value in a finite set {1,2,⋯,M}, M is the total number of dynamic models, $F_{k-1}(x_{k-1},r_{k})$ and $H_{k}(x_{k},r_{k})$ are the state transition function and measurement function based on the mode $r_{k}$ respectively, $w_{k-1}(r_{k})$ is the process noise vector based on the mode $r_{k}$, and $\varepsilon_{k}$ is the measurement noise vector. The mode $r_{k}$ is assumed to be a homogeneous Markov chain with the transition probability $\pi_{ij}=p(r_{k}=j|r_{k-1}=i)$, and $w_{k-1}(r_{k})$ is assumed to be uncorrelated zero-mean Gaussian noises with covariance matrix $Q_{k-1}(r_{k})$. Throughout this paper, we abbreviate $F_{k-1}(x_{k-1},r_{k}=i)$, $H_{k}(x_{k},r_{k}=i)$, $w_{k-1}(r_{k}=i)$ and $Q_{k-1}(r_{k}=i)$ by $F_{k-1}^{i}(x_{k-1})$, $H_{k}^{i}(x_{k})$, $w_{k-1}^{i}$ and $Q_{k-1}^{i}$, respectively. Then, the transition probability density function (PDF) of state is given by $p(x_{k}|x_{k-1},r_{k}=i)=N(x_{k};F_{k-1}^{i}(x_{k-1}),Q_{k-1}^{i})$, where N(·;m,P) denotes a Gaussian PDF with mean m and covariance matrix P.

When measurement outliers occur, the distribution of measurement noises has heavy-tailed non-Gaussian characteristic. The performance of traditional IMM filter based on Gaussian noise assumption may dramatically degrade since it is very sensitive to heavy-tailed noises. Since the Student’s t-distribution has heavier tail and is more robust to outliers than Gaussian distribution, we model the measurement noises as Student’s t-distribution, i.e., $\varepsilon_{k}∼\mathrm{Std}(\varepsilon_{k};0,R_{k},v_{k})$, where Std(·;μ,R,v) stands for a Student’s *t* PDF with mean μ, scale matrix R and dof v. The Student’s *t*-distribution can be represented by an infinite mixture of Gaussian distributions as [32]

$$\mathrm{Std}(x;\mu ,R,v)=\int_{0}^{\infty }N(x;\mu ,R/\lambda )G(\lambda ;v/2,v/2)d\lambda$$

where λ is an auxiliary random variable, G(·;a,b) denotes a Gamma PDF with shape parameter a and rate parameter b. The expression for G(·;a,b) is

(1) $$G(x;a,b)=\frac{b^{a}}{\Gamma (a)}x^{a-1}\exp(-bx)\;x\geq 0$$

where $\Gamma (a)=\int_{0}^{+\infty }\exp(-t)t^{a-1}dt$ is the Gamma function. Then, the likelihood PDF conditioned on the mode $r_{k}=i$ can be written as

(2) $$\begin{array}{ll} p(z_{k}|x_{k},r_{k}=i)= & \mathrm{Std}(z_{k};H_{k}^{i}(x_{k}),R_{k},v_{k})\\ & \int_{0}^{\infty }N(z_{k};H_{k}^{i}(x_{k}),R_{k}/\lambda_{k})G(\lambda_{k};v_{k}/2,v_{k}/2)d\lambda_{k} \end{array}$$

The tail thickness of Student’s *t*-distribution is controlled by the dof. When the dof decreases, the tail becomes thicker. When the dof goes to infinity, the Student’s *t*-distribution approaches a Gaussian distribution. Shen et al. [31] assumed that the posterior distribution of the auxiliary variable $\lambda_{k}$ is a Gamma distribution, i.e., $p(\lambda_{k}|r_{k}=i,z_{1:k})=G(\lambda_{k};{\hat{\alpha }}_{k}^{i},{\hat{\beta }}_{k}^{i})$ and used the VB approach to estimate the parameters ${\hat{\alpha }}_{k}^{i}$ and ${\hat{\beta }}_{k}^{i}$. However, the estimates of both ${\hat{\alpha }}_{k}^{i}$ and ${\hat{\beta }}_{k}^{i}$ which determine the dof of Student’s *t*-distribution become large after several filtering steps. Thus, the distribution of measurement noises loses the heavy-tailed property and the robustness of the filter cannot be guaranteed. To prevent the growth of the dof estimates, we assume that the posterior distribution of the dof $v_{k}$ is a Gamma distribution, i.e., $p(v_{k}|r_{k}=i,z_{1:k})=G(v_{k};{\hat{a}}_{k}^{i},{\hat{b}}_{k}^{i})$, and estimate the parameters ${\hat{a}}_{k}^{i}$ and ${\hat{b}}_{k}^{i}$ instead. Furthermore, in order to deal with the uncertainty of scale matrix $R_{k}$, we choose inverse Wishart distribution as the posterior distribution of $R_{k}$, i.e., $p(R_{k}|r_{k}=i,z_{1:k})=\mathrm{IW}(R_{k};{\hat{t}}_{k}^{i},T_{k}^{i})$, where IW(·;t,T) stands for an inverse Wishart PDF with degree t and inverse scale matrix T. The PDF of inverse Wishart distribution is defined by

(3) $$\mathrm{IW}(R;t,T)=\frac{\det{\left(T\right)}^{0.5t}}{2^{0.5tm}\Gamma_{m}(0.5t)}\det{\left(R\right)}^{-0.5(t+m+1)}\exp(-0.5\mathrm{tr}(TR^{-1}))$$

where $\Gamma_{m}(0.5t)=\pi^{0.25m(m-1)}\prod_{i=0}^{m-1}\Gamma (0.5(t-i))$ Then, the unknown statistic of the scale matrix $R_{k}$ can be determined by estimating the parameters ${\hat{t}}_{k}^{i}$ and $T_{k}^{i}$.

## 3. Design of Robust IMM Filter

### 3.1. Model Interaction

We assume that the mode-conditioned posterior distributions of the state $x_{k-1}$, the scale matrix $R_{k-1}$ and the dof $v_{k-1}$ at time k−1 are Gaussian, inverse Wishart, and Gamma distributions, respectively, and that they are mutually independent. Then, the joint mode-conditioned posterior PDF of $x_{k-1}$, $R_{k-1}$ and $v_{k-1}$ can be expressed as

$$\begin{array}{ll} p(x_{k-1},R_{k-1},v_{k-1}|r_{k-1}=j,z_{1:k-1}) & =p(x_{k-1}|r_{k-1}=j,z_{1:k-1})p(R_{k-1}|r_{k-1}=j,z_{1:k-1})p(v_{k-1}|r_{k-1}=j,z_{1:k-1})\\ & =N(x_{k-1};{\hat{x}}_{k-1}^{j},P_{k-1}^{j})\mathrm{IW}(R_{k-1};{\hat{t}}_{k-1}^{j},T_{k-1}^{j})G(v_{k-1};{\hat{a}}_{k-1}^{j},{\hat{b}}_{k-1}^{j}) \end{array}$$

Let $u_{k}^{i}=p(r_{k}=i|z_{1:k})$ and $u_{k|k-1}^{i}=p(r_{k}=i|z_{1:k-1})$ denote the posterior and predicted mode probabilities respectively, and $u_{k-1}^{ij}=p(r_{k-1}=i|r_{k}=j,z_{1:k-1})$ denote the mixing probability, we have

(4) $$\begin{array}{ll} u_{k|k-1}^{j} & =\sum_{i=1}^{M}p(r_{k}=j|r_{k-1}=i,z_{1:k-1})p(r_{k-1}=i|z_{1:k-1})\\ & =\sum_{i=1}^{M}\pi_{ij}u_{k-1}^{i} \end{array}$$

(5) $$\begin{array}{ll} u_{k-1}^{ij} & =\frac{p(r_{k}=j|r_{k-1}=i,z_{1:k-1})p(r_{k-1}=i|z_{1:k-1})}{p(r_{k}=j|z_{1:k-1})}\\ & =\pi_{ij}u_{k-1}^{i}/u_{k|k-1}^{j} \end{array}$$

The mixing PDF of $x_{k-1}$, $R_{k-1}$ and $v_{k-1}$ is given by

(6) $$\begin{array}{ll} p(x_{k-1},R_{k-1},v_{k-1}|r_{k}=i,z_{1:k-1}) & =\sum_{j=1}^{M}p(x_{k-1},R_{k-1},v_{k-1}|r_{k-1}=j,z_{1:k-1})p(r_{k-1}=j|r_{k}=i,z_{1:k-1})\\ & =\sum_{j=1}^{M}u_{k-1}^{ji}N(x_{k-1};{\hat{x}}_{k-1}^{j},P_{k-1}^{j})\mathrm{IW}(R_{k-1};{\hat{t}}_{k-1}^{j},T_{k-1}^{j})G(v_{k-1};{\hat{a}}_{k-1}^{j},{\hat{b}}_{k-1}^{j}) \end{array}$$

We approximate the above summed PDF in the right side of (6) by a single one as

(7) $$p(x_{k-1},R_{k-1},v_{k-1}|r_{k}=i,z_{1:k-1})=N(x_{k-1};{\hat{x}}_{k-1}^{0i},P_{k-1}^{0i})\mathrm{IW}(R_{k-1};{\hat{t}}_{k-1}^{0i},T_{k-1}^{0i})G(v_{k-1};{\hat{a}}_{k-1}^{0i},{\hat{b}}_{k-1}^{0i})$$

By matching the first and the second-order moments between (6) and (7) for the state distribution, we obtain the mixing state ${\hat{x}}_{k-1}^{0i}$ and the covariance matrix $P_{k-1}^{0i}$ as

(8) $${\hat{x}}_{k-1}^{0i}=\sum_{j=1}^{M}u_{k-1}^{ji}{\hat{x}}_{k-1}^{j}$$

(9) $$P_{k-1}^{0i}=\sum_{j=1}^{M}u_{k-1}^{ji}(P_{k-1}^{j}+({\hat{x}}_{k-1}^{j}-{\hat{x}}_{k-1}^{0i}){\left({\hat{x}}_{k-1}^{j}-{\hat{x}}_{k-1}^{0i}\right)}^{T})$$

However, the mixing parameters ${\hat{t}}_{k-1}^{0i}$ and $T_{k-1}^{0i}$ cannot be computed analytically by matching the first two moments of inverse Wishart distributions between (6) and (7) due to the complexity of second-order moment. We adopt the method of minimizing weighted Kullback–Leibler (KL) divergence in [33] to overcome this difficulty and derive that

(10) $${\hat{t}}_{k-1}^{0i}=\sum_{j=1}^{M}u_{k-1}^{ji}{\hat{t}}_{k-1}^{j}$$

(11) $$T_{k-1}^{0i}=\sum_{j=1}^{M}u_{k-1}^{ji}T_{k-1}^{j}$$

For the Gamma distribution, we also use the method of matching the first two moments like the state. According to (6), the conditional mean and covariance of $v_{k-1}$ are given by

$$\begin{array}{ll} E(v_{k-1}|r_{k}=i,z_{1:k-1}) & =\int v_{k-1}p(v_{k-1}|r_{k}=i,z_{1:k-1})dv_{k-1}\\ & =\int v_{k-1}\sum_{j=1}^{M}u_{k-1}^{ji}G(v_{k-1};{\hat{a}}_{k-1}^{j},{\hat{b}}_{k-1}^{j})dv_{k-1}\\ & =\sum_{j=1}^{M}u_{k-1}^{ji}\int v_{k-1}G(v_{k-1};{\hat{a}}_{k-1}^{j},{\hat{b}}_{k-1}^{j})dv_{k-1} \end{array}$$

$$\begin{array}{ll} \mathrm{Var}(v_{k-1}|r_{k}=i,z_{1:k-1}) & =\int {\left(v_{k-1}-E(v_{k-1}|r_{k}=i,z_{1:k-1})\right)}^{2}p(v_{k-1}|r_{k}=i,z_{1:k-1})dv_{k-1}\\ & =\int {\left(v_{k-1}-E(v_{k-1}|r_{k}=i,z_{1:k-1})\right)}^{2}\sum_{j=1}^{M}u_{k-1}^{ji}G(v_{k-1};{\hat{a}}_{k-1}^{j},{\hat{b}}_{k-1}^{j})dv_{k-1}\\ & =\sum_{j=1}^{M}u_{k-1}^{ji}\int {\left(v_{k-1}-E(v_{k-1}|r_{k}=i,z_{1:k-1})\right)}^{2}G(v_{k-1};{\hat{a}}_{k-1}^{j},{\hat{b}}_{k-1}^{j})dv_{k-1} \end{array}$$

Since the mean and covariance of a Gamma PDF G(·;a,b) are a/b and $a/b^{2}$ respectively, we have

(12) $$E(v_{k-1}|r_{k}=i,z_{1:k-1})=\sum_{j=1}^{M}u_{k-1}^{ji}{\hat{a}}_{k-1}^{j}/{\hat{b}}_{k-1}^{j}$$

(13) $$\begin{array}{l} \mathrm{var}(v_{k-1}|r_{k}=i,z_{1:k-1})=\\ \sum_{j=1}^{M}u_{k-1}^{ji}\int \{{\left(v_{k-1}-{\hat{a}}_{k-1}^{j}/{\hat{b}}_{k-1}^{j}\right)}^{2}+{\left({\hat{a}}_{k-1}^{j}/{\hat{b}}_{k-1}^{j}-E(v_{k-1}|r_{k}=i,z_{1:k-1})\right)}^{2}\}G(v_{k-1};{\hat{a}}_{k-1}^{j},{\hat{b}}_{k-1}^{j})dv_{k-1}\\ =\sum_{j=1}^{M}u_{k-1}^{ji}({\hat{a}}_{k-1}^{j}/{\left({\hat{b}}_{k-1}^{j}\right)}^{2}+{\left({\hat{a}}_{k-1}^{j}/{\hat{b}}_{k-1}^{j}-\sum_{j=1}^{M}u_{k-1}^{ji}{\hat{a}}_{k-1}^{j}/{\hat{b}}_{k-1}^{j}\right)}^{2}) \end{array}$$

On the other hand, according to (7), we have

(14) $$E(v_{k-1}|r_{k}=i,z_{1:k-1})={\hat{a}}_{k-1}^{0i}/{\hat{b}}_{k-1}^{0i},$$

(15) $$\mathrm{Var}(v_{k-1}|r_{k}=i,z_{1:k-1})={\hat{a}}_{k-1}^{0i}/{\left({\hat{b}}_{k-1}^{0i}\right)}^{2}$$

By combining (12)–(15), the mixing parameters ${\hat{a}}_{k-1}^{0i}$ and ${\hat{b}}_{k-1}^{0i}$ for the Gamma distribution can be calculated as

(16) $${\hat{a}}_{k-1}^{0i}=\frac{{\left(\sum_{j=1}^{M}u_{k-1}^{ji}{\hat{a}}_{k-1}^{j}/{\hat{b}}_{k-1}^{j}\right)}^{2}}{\sum_{j=1}^{M}u_{k-1}^{ji}({\hat{a}}_{k-1}^{j}/{\left({\hat{b}}_{k-1}^{j}\right)}^{2}+{\left({\hat{a}}_{k-1}^{j}/{\hat{b}}_{k-1}^{j}-\sum_{j=1}^{M}u_{k-1}^{ji}{\hat{a}}_{k-1}^{j}/{\hat{b}}_{k-1}^{j}\right)}^{2})},$$

(17) $${\hat{b}}_{k-1}^{0i}=\frac{\sum_{j=1}^{M}u_{k-1}^{ji}{\hat{a}}_{k-1}^{j}/{\hat{b}}_{k-1}^{j}}{\sum_{j=1}^{M}u_{k-1}^{ji}({\hat{a}}_{k-1}^{j}/{\left({\hat{b}}_{k-1}^{j}\right)}^{2}+{\left({\hat{a}}_{k-1}^{j}/{\hat{b}}_{k-1}^{j}-\sum_{j=1}^{M}u_{k-1}^{ji}{\hat{a}}_{k-1}^{j}/{\hat{b}}_{k-1}^{j}\right)}^{2})}\;.$$

### 3.2. Time Prediction

The time prediction step is to derive the mode-conditioned predicted PDF $p(x_{k},R_{k},v_{k}|r_{k}=i,z_{1:k-1})$ at time k by using the mixing PDF $p(x_{k-1},R_{k-1},v_{k-1}|r_{k}=i,z_{1:k-1})$ and the dynamical models at time k−1.

The mode-conditioned predicted PDF can be calculated by the following Chapman–Kolmogorov equation:

(18) $$\begin{array}{l} p(x_{k},R_{k},v_{k}|r_{k}=i,z_{1:k-1})=\\ \int p(x_{k-1},R_{k-1},v_{k-1}|r_{k}=i,z_{1:k-1})p(x_{k},R_{k},v_{k}|x_{k-1},R_{k-1},v_{k-1},r_{k}=i)dx_{k-1}dR_{k-1}dv_{k-1} \end{array}.$$

We assume that the dynamical models of the state, the scale matrix and the dof are independent, i.e.,

(19) $$p(x_{k},R_{k},v_{k}|x_{k-1},R_{k-1},v_{k-1},r_{k})=p(x_{k}|x_{k-1},r_{k})p(R_{k}|R_{k-1},r_{k})p(v_{k}|v_{k-1},r_{k}).$$

According to (7), (18) and (19), we have

(20) $$\begin{array}{l} p(x_{k},R_{k},v_{k}|r_{k}=i,z_{1:k-1})=\int N(x_{k};F_{k-1}^{i}(x_{k-1}),Q_{k-1}^{i})N(x_{k-1};{\hat{x}}_{k-1}^{0i},P_{k-1}^{0i})dx_{k-1}\times \\ \int p(R_{k}|R_{k-1},r_{k})\mathrm{IW}(R_{k-1};{\hat{t}}_{k-1}^{0i},T_{k-1}^{0i})dR_{k-1}\int p(v_{k}|v_{k-1},r_{k})G(v_{k-1};{\hat{a}}_{k-1}^{0i},{\hat{b}}_{k-1}^{0i})dv_{k-1} \end{array}.$$

We can see that the mode-conditioned predicted distributions of the state, the scale matrix and the dof are also independent. These distributions are assumed to be Gaussian, inverse Wishart and Gamma distributions, respectively, which have the same form of PDF as the posterior distributions. Then, the mode-conditioned predicted PDF can be represented by

(21) $$p(x_{k},R_{k},v_{k}|r_{k}=i,z_{1:k-1})=N(x_{k};{\hat{x}}_{k|k-1}^{i},P_{k|k-1}^{i})\mathrm{IW}(R_{k};{\hat{t}}_{k|k-1}^{i},T_{k|k-1}^{i})G(v_{k};{\hat{a}}_{k|k-1}^{i},{\hat{b}}_{k|k-1}^{i}).$$

By matching the first and the second-order moments between (20) and (21) for the state, we obtain the predicted mean and the covariance matrix as

(22) $${\hat{x}}_{k|k-1}^{i}=\int F_{k-1}^{i}(x_{k-1})N(x_{k-1};{\hat{x}}_{k-1}^{0i},P_{k-1}^{0i})dx_{k-1},$$

(23) $$P_{k|k-1}^{i}=\int (F_{k-1}^{i}(x_{k-1})-{\hat{x}}_{k|k-1}^{i}){\left(F_{k-1}^{i}(x_{k-1})-{\hat{x}}_{k|k-1}^{i}\right)}^{T}N(x_{k-1};{\hat{x}}_{k-1}^{0i},P_{k-1}^{0i})dx_{k-1}+Q_{k-1}^{i}$$

However, it is hard to compute the predicted parameters of inverse Wishart and Gamma distributions since the dynamical models of the scale matrix and the dof are usually unknown in practice. Here, a heuristic dynamical model is chosen as in [34] by introducing a forgetting factor ρ∈(0,1], which indicates the extent of parameter fluctuation. Then, the predicted parameters are given by

(24) $$T_{k|k-1}^{i}=\rho T_{k-1}^{0i},$$

(25) $${\hat{t}}_{k|k-1}^{i}=\rho ({\hat{t}}_{k-1}^{0i}-m-1)+m+1,$$

(26) $${\hat{a}}_{k|k-1}^{i}=\rho {\hat{a}}_{k-1}^{0i},$$

(27) $${\hat{b}}_{k|k-1}^{i}=\rho {\hat{b}}_{k-1}^{0i}$$

When ρ=1, the parameters are kept stationary. When ρ is close to 0, the parameters are high time-fluctuation.

### 3.3. Measurement Update

We define $\Theta =\{x_{k},R_{k},\lambda_{k},v_{k}\}$. The measurement update step is to compute the mode-conditioned posterior PDF $p(\Theta |z_{1:k},r_{k}=i)$ and the posterior mode probability $u_{k}^{i}$ when a new measurement $z_{k}$ is collected at time k. However, deriving the mode-conditioned posterior PDF is analytically intractable. We employ the VB approach [35] to obtain an approximated solution.

According to the VB approach, the mode-conditioned posterior PDF is approximated by a free form factored PDF:

$$p(\Theta |z_{1:k},r_{k}=i)\approx q^{i}(x_{k})q^{i}(R_{k})q^{i}(\lambda_{k})q^{i}(v_{k})$$

The posterior PDFs $q^{i}(x_{k})$, $q^{i}(R_{k})$, $q^{i}(\lambda_{k})$ and $q^{i}(v_{k})$ are calculated by minimizing the KL divergence as

$$\underset{q^{i}(x_{k}),q^{i}(R_{k}),q^{i}(\lambda_{k}),q^{i}(v_{k})}{\min}\mathrm{KL}(q^{i}(x_{k})q^{i}(R_{k})q^{i}(\lambda_{k})q^{i}(v_{k})||p(\Theta |z_{1:k},r_{k}=i)),$$

where KL(q(·)||p(·))≜∫q(x)ln(q(x)/p(x))dx is the KL divergence. The solutions of the above optimal problem satisfy

(28) $$\ln q^{i}(x_{k})=E_{q^{i}(R_{k})q^{i}(\lambda_{k})q^{i}(v_{k})}\ln p(\Theta ,z_{1:k}|r_{k}=i)+c_{x},$$

(29) $$\ln q^{i}(R_{k})=E_{q^{i}(x_{k})q^{i}(\lambda_{k})q^{i}(v_{k})}\ln p(\Theta ,z_{1:k}|r_{k}=i)+c_{R},$$

(30) $$\ln q^{i}(\lambda_{k})=E_{q^{i}(x_{k})q^{i}(R_{k})q^{i}(v_{k})}\ln p(\Theta ,z_{1:k}|r_{k}=i)+c_{\lambda },$$

(31) $$\ln q^{i}(v_{k})=E_{q^{i}(x_{k})q^{i}(R_{k})q^{i}(\lambda_{k})}\ln p(\Theta ,z_{1:k}|r_{k}=i)+c_{v}$$

where $c_{x}$, $c_{R}$, $c_{\lambda }$ and $c_{v}$ are constants independent of $x_{k}$, $R_{k}$, $\lambda_{k}$ and $v_{k}$, respectively.

According to (2) and (21), we have

(32) $$\begin{array}{ll} p(\Theta ,z_{1:k}|r_{k}=i) & =p(z_{1:k-1}|r_{k}=i)p(\Theta ,z_{k}|r_{k}=i,z_{1:k-1})\\ & =p(z_{1:k-1}|r_{k}=i)p(x_{k}|z_{1:k-1},r_{k}=i)p(z_{k}|x_{k},R_{k},\lambda_{k},r_{k}=i)\\ & \;\times p(R_{k}|z_{1:k-1},r_{k}=i)p(\lambda_{k}|v_{k})p(v_{k}|z_{1:k-1},r_{k}=i)\\ & =p(z_{1:k-1}|r_{k}=i)N(x_{k};{\hat{x}}_{k|k-1}^{i},P_{k|k-1}^{i})N(z_{k};H_{k}^{i}(x_{k}),R_{k}/\lambda_{k})\\ & \;\times \mathrm{IW}(R_{k};{\hat{t}}_{k|k-1}^{i},T_{k|k-1}^{i})G(\lambda_{k};v_{k}/2,v_{k}/2)G(v_{k};{\hat{a}}_{k|k-1}^{i},{\hat{b}}_{k|k-1}^{i}) \end{array}.$$

Substituting (1), (3) and the expression of Gaussian PDF into (32), we obtain the detailed expression of $p(\Theta ,z_{1:k}|r_{k}=i)$

(33) $$\begin{array}{ll} \ln p(\Theta ,z_{1:k}|r_{k}=i)= & -0.5{\left(x_{k}-{\hat{x}}_{k|k-1}^{i}\right)}^{T}{\left(P_{k|k-1}^{i}\right)}^{-1}(x_{k}-{\hat{x}}_{k|k-1}^{i})-\\ & 0.5\lambda_{k}{\left(z_{k}-H_{k}^{i}(x_{k})\right)}^{T}R_{k}^{-1}(z_{k}-H_{k}^{i}(x_{k}))+\\ & 0.5(m+v_{k}-2)\ln\lambda_{k}-0.5v_{k}\lambda_{k}+({\hat{a}}_{k|k-1}^{i}-1)\ln v_{k}-{\hat{b}}_{k|k-1}^{i}v_{k}+0.5v_{k}\ln(0.5v_{k})\\ & -\ln\Gamma (0.5v_{k})-0.5({\hat{t}}_{k|k-1}^{i}+m+2)\ln\det(R_{k})-0.5\mathrm{tr}(T_{k|k-1}^{i}R_{k}^{-1})+c \end{array},$$

where c denotes a constant with respect to Θ.

Substituting (33) into (28) yields

$$\ln q^{i}(x_{k})=-0.5{\left(x_{k}-{\hat{x}}_{k|k-1}^{i}\right)}^{T}{\left(P_{k|k-1}^{i}\right)}^{-1}(x_{k}-{\hat{x}}_{k|k-1}^{i})-0.5{\left(z_{k}-H_{k}^{i}(x_{k})\right)}^{T}{\left({\tilde{R}}_{k}\right)}^{-1}(z_{k}-H_{k}^{i}(x_{k}))+c_{x},$$

where ${\tilde{R}}_{k}$ is given by

(34) $${\tilde{R}}_{k}={\left(E_{q^{i}(\lambda_{k})}\lambda_{k}\right)}^{-1}{\left(E_{q^{i}(R_{k})}R_{k}^{-1}\right)}^{-1},$$

where $E_{q^{i}(\cdot )}$ denotes the expectation with respect to the PDF $q^{i}(\cdot )$. ${\tilde{R}}_{k}$ can be deemed as modified covariance matrix of measurements taking into account the uncertainty of both and $R_{k}$ and $\lambda_{k}$. Then,

$$q^{i}(x_{k})\propto N(x_{k};{\hat{x}}_{k|k-1}^{i},P_{k|k-1}^{i})N(z_{k};H_{k}^{i}(x_{k}),{\tilde{R}}_{k}).$$

According to the construction of nonlinear Gaussian filter in [36], $q^{i}(x_{k})$ can be approximated by a Gaussian PDF, i.e.,

(35) $$q^{i}(x_{k})\approx N(x_{k};{\hat{x}}_{k}^{i},P_{k}^{i}),$$

where

(36) $${\hat{x}}_{k}^{i}={\hat{x}}_{k|k-1}^{i}+P_{xz,k|k-1}^{i}{\left(P_{zz,k|k-1}^{i}\right)}^{-1}(z_{k}-{\hat{z}}_{k|k-1}),$$

(37) $${\hat{z}}_{k|k-1}^{i}=\int H_{k}^{i}(x_{k})N(x_{k};{\hat{x}}_{k|k-1}^{i},P_{k|k-1}^{i})dx_{k},$$

(38) $$P_{zz,k|k-1}^{i}=\int (H_{k}^{i}(x_{k})-{\hat{z}}_{k|k-1}^{i}){\left(H_{k}^{i}(x_{k})-{\hat{z}}_{k|k-1}^{i}\right)}^{T}N(x_{k};{\hat{x}}_{k|k-1}^{i},P_{k|k-1}^{i})dx_{k}+{\tilde{R}}_{k},$$

(39) $$P_{xz,k|k-1}^{i}=\int (x_{k}-{\hat{x}}_{k|k-1}^{i}){\left(H_{k}^{i}(x_{k})-{\hat{z}}_{k|k-1}^{i}\right)}^{T}N(x_{k},{\hat{x}}_{k|k-1}^{i},P_{k|k-1}^{i})dx_{k},$$

(40) $$P_{k}^{i}=P_{k|k-1}^{i}-P_{xz,k|k-1}^{i}{\left(P_{zz,k|k-1}^{i}\right)}^{-1}{\left(P_{xz,k|k-1}^{i}\right)}^{T}$$

Substituting (33) into (29) yields

$$\ln q^{i}(R_{k})=-0.5\mathrm{tr}(\{(E_{q^{i}(\lambda_{k})}\lambda_{k})A_{k}^{i}+T_{k|k-1}^{i}\}R_{k}^{-1})-0.5({\hat{t}}_{k|k-1}^{i}+m+2)\ln\det(R_{k})+c_{R},$$

where

(41) $$A_{k}^{i}=E_{q^{i}(x_{k})}(z_{k}-H_{k}^{i}(x_{k})){\left(z_{k}-H_{k}^{i}(x_{k})\right)}^{T}$$

According to (3), we can deduce that $q^{i}(R_{k})$ is an inverse Wishart PDF with the following expression:

(42) $$q^{i}(R_{k})=\mathrm{IW}(R_{k};{\hat{t}}_{k}^{i},T_{k}^{i}),$$

where

(43) $${\hat{t}}_{k}^{i}={\hat{t}}_{k|k-1}^{i}+1,$$

(44) $$T_{k}^{i}=(E_{q^{i}(\lambda_{k})}\lambda_{k})A_{k}^{i}+T_{k|k-1}^{i}$$

Substituting (33) into (30) yields

$$\ln q^{i}(\lambda_{k})=-0.5(\mathrm{tr}((E_{q^{i}(R_{k})}R_{k}^{-1})A_{k}^{i})+E_{q^{i}(v_{k})}v_{k})\lambda_{k}+0.5(m+E_{q^{i}(v_{k})}v_{k}-2)\ln\lambda_{k}+c_{\lambda }.$$

According to (1), $q^{i}(\lambda_{k})$ is a Gamma PDF as

(45) $$q^{i}(\lambda_{k})=G(\lambda_{k};{\hat{\alpha }}_{k}^{i},{\hat{\beta }}_{k}^{i}),$$

where

(46) $${\hat{\alpha }}_{k}^{i}=0.5(m+E_{q^{i}(v_{k})}v_{k}),$$

(47) $${\hat{\beta }}_{k}^{i}=0.5(\mathrm{tr}((E_{q^{i}(R_{k})}R_{k}^{-1})A_{k}^{i}))+E_{q^{i}(v_{k})}v_{k})$$

Substituting (33) into (31), and using the Stirling’s approximation $\ln\Gamma (0.5v_{k})\approx 0.5(v_{k}-1)\ln0.5v_{k}-0.5v_{k}$, we obtain

$$\ln q^{i}(v_{k})=({\hat{a}}_{k|k-1}^{i}-0.5)\ln v_{k}+(0.5E_{q^{i}(\lambda_{k})}\ln\lambda_{k}-0.5E_{q^{i}(\lambda_{k})}\lambda_{k}-{\hat{b}}_{k|k-1}^{i}+0.5)v_{k}+c_{v}$$

According to (1), $q^{i}(v_{k})$ is also a Gamma PDF as

(48) $$q^{i}(v_{k})=G(v_{k};{\hat{a}}_{k}^{i},{\hat{b}}_{k}^{i}),$$

where

(49) $${\hat{a}}_{k}^{i}={\hat{a}}_{k|k-1}^{i}+0.5,$$

(50) $${\hat{b}}_{k}^{i}={\hat{b}}_{k|k-1}^{i}-0.5E_{q^{i}(\lambda_{k})}\ln\lambda_{k}+0.5E_{q^{i}(\lambda_{k})}\lambda_{k}-0.5$$

The unknown expectations in (34), (41), (44), (46), (47) and (50) are given by

(51) $$E_{q^{i}(\lambda_{k})}\lambda_{k}={\hat{\alpha }}_{k}^{i}/{\hat{\beta }}_{k}^{i},$$

(52) $$E_{q^{i}(R_{k})}R_{k}^{-1}=({\hat{t}}_{k}^{i}-m-1)(T_{k}^{i}{)}^{-1},$$

(53) $$\begin{array}{ll} A_{k}^{i} & =\int (z_{k}-H_{k}^{i}(x_{k})){\left(z_{k}-H_{k}^{i}(x_{k})\right)}^{T}N(x_{k};{\hat{x}}_{k}^{i},P_{k}^{i})dx_{k}\\ & =(z_{k}-{\hat{z}}_{k}^{i}){\left(z_{k}-{\hat{z}}_{k}^{i}\right)}^{T}+\int (H_{k}^{i}(x_{k})-{\hat{z}}_{k}^{i}){\left(H_{k}^{i}(x_{k})-{\hat{z}}_{k}^{i}\right)}^{T}N(x_{k};{\hat{x}}_{k}^{i},P_{k}^{i})dx_{k} \end{array}$$

(54) $${\hat{z}}_{k}^{i}=\int H_{k}^{i}(x_{k})N(x_{k};{\hat{x}}_{k}^{i},P_{k}^{i})dx_{k},$$

(55) $$E_{q^{i}(v_{k})}v_{k}={\hat{a}}_{k}^{i}/{\hat{b}}_{k}^{i},$$

(56) $$E_{q^{i}(\lambda_{k})}\ln\lambda_{k}=\psi ({\hat{\alpha }}_{k}^{i})-\ln{\hat{\beta }}_{k}^{i}$$

where ψ(x) is the digamma function.

Combining (36)–(40), (43), (44), (46), (47) and (49)–(56), the fixed point iteration algorithm can be employed to acquire the unknown quantities ${\hat{x}}_{k}^{i}$, $P_{k}^{i}$, ${\hat{t}}_{k}^{i}$, $T_{k}^{i}$, ${\hat{\alpha }}_{k}^{i}$, ${\hat{\beta }}_{k}^{i}$, ${\hat{a}}_{k}^{i}$, and ${\hat{b}}_{k}^{i}$. The iteration is terminated when the variations of these quantities are small enough.

The posterior mode probability can be calculated by

(57) $$\begin{array}{ll} u_{k}^{i} & =\frac{p(r_{k}=i|z_{1:k-1})p(z_{k}|r_{k}=i,z_{1:k-1})}{p(z_{k}|z_{1:k-1})}\\ & =\frac{u_{k|k-1}^{i}p(z_{k}|r_{k}=i,z_{1:k-1})}{\sum_{i=1}^{M}u_{k|k-1}^{i}p(z_{k}|r_{k}=i,z_{1:k-1})} \end{array}$$

In (57), the predicted mode probability $u_{k|k-1}^{i}$ is given by (4), and the predicted likelihood $p(z_{k}|r_{k}=i,z_{1:k-1})$ can be derived by

$$p(z_{k}|r_{k}=i,z_{1:k-1})=\int p(\Theta ,z_{k}|r_{k}=i,z_{1:k-1})d\Theta$$

However, the above integration is computationally infeasible since $x_{k}$, $R_{k}$, $\lambda_{k}$ and $v_{k}$ are coupled in $p(\Theta ,z_{k}|r_{k}=i,z_{1:k-1})$ according to (32). We use the similar variational approach in [37] to derive an approximated predicted likelihood. The predicted likelihood can be rewritten as

$$\begin{array}{ll} \ln p(z_{k}|r_{k}=i,z_{1:k-1})= & \int q^{i}(x_{k})q^{i}(R_{k})q^{i}(\lambda_{k})q^{i}(v_{k})\ln\frac{p(\Theta ,z_{k}|r_{k}=i,z_{1:k-1})}{q^{i}(x_{k})q^{i}(R_{k})q^{i}(\lambda_{k})q^{i}(v_{k})}dx_{k}dR_{k}d\lambda_{k}dv_{k}\\ & +\mathrm{KL}(q^{i}(x_{k})q^{i}(R_{k})q^{i}(\lambda_{k})q^{i}(v_{k})||p(\Theta |r_{k}=i,z_{1:k})) \end{array}$$

Since the KL divergence term in the right side of the above equation is minimized by VB approach, the predicted likelihood can be approximated by

(58) $$\ln p(z_{k}|r_{k}=i,z_{1:k-1})\approx \int q^{i}(x_{k})q^{i}(R_{k})q^{i}(\lambda_{k})q^{i}(v_{k})\ln\frac{p(\Theta ,z_{k}|r_{k}=i,z_{1:k-1})}{q^{i}(x_{k})q^{i}(R_{k})q^{i}(\lambda_{k})q^{i}(v_{k})}dx_{k}dR_{k}d\lambda_{k}dv_{k}$$

Substituting (35), (42), (45), (48) and the expression of $p(\Theta ,z_{k}|r_{k}=i,z_{1:k-1})$ in (32) into (58) yields

(59) $$\begin{array}{l} \ln p(z_{k}|r_{k}=i,z_{1:k-1})\approx 0.5\{\ln\det(P_{k}^{i})-\ln\det(P_{k|k-1}^{i})+n-m\ln\pi +m\psi ({\hat{\alpha }}_{k}^{i})\\ -m\ln{\hat{\beta }}_{k}^{i}-({\hat{\alpha }}_{k}^{i}/{\hat{\beta }}_{k}^{i})\mathrm{tr}\{({\hat{t}}_{k}^{i}-m-1){\left(T_{k}^{i}\right)}^{-1}A_{k}^{i}\}+{\hat{t}}_{k|k-1}^{i}\ln\det(T_{k|k-1}^{i})-{\hat{t}}_{k}^{i}\ln\det(T_{k}^{i})\\ +2\ln\Gamma_{m}(0.5{\hat{t}}_{k}^{i})-2\ln\Gamma_{m}(0.5{\hat{t}}_{k|k-1}^{i})-\mathrm{tr}({\left(P_{k|k-1}^{i}\right)}^{-1}\{P_{k}^{i}+({\hat{x}}_{k}^{i}-{\hat{x}}_{k|k-1}^{i}){\left({\hat{x}}_{k}^{i}-{\hat{x}}_{k|k-1}^{i}\right)}^{T}\})\\ +\mathrm{tr}(({\hat{t}}_{k}^{i}-m-1)(T_{k}^{i}-T_{k|k-1}^{i}){\left(T_{k}^{i}\right)}^{-1})+2{\hat{a}}_{k|k-1}^{i}\ln{\hat{b}}_{k|k-1}^{i}-2({\hat{a}}_{k}^{i}-1)\ln{\hat{b}}_{k}^{i}+2\ln\Gamma ({\hat{a}}_{k}^{i})\\ -2\ln\Gamma ({\hat{a}}_{k|k-1}^{i})+2({\hat{b}}_{k}^{i}-{\hat{b}}_{k|k-1}^{i}){\hat{a}}_{k}^{i}/{\hat{b}}_{k}^{i}-2\psi ({\hat{a}}_{k}^{i})\} \end{array}$$

The final estimate of the state is given by a probabilistically weighted average of all the mode-conditioned estimates, i.e.,

(60) $${\hat{x}}_{k}=\sum_{i=1}^{M}u_{k}^{i}{\hat{x}}_{k}^{i},$$

and the corresponding covariance matrix is calculated as

(61) $$P_{k}=\sum_{i=1}^{M}u_{k}^{i}(P_{k}^{i}+({\hat{x}}_{k}^{i}-{\hat{x}}_{k}){\left({\hat{x}}_{k}^{i}-{\hat{x}}_{k}\right)}^{T}).$$

### 3.4. Approximated Gaussian Integrations Based on Unscented Transform

Due to the nonlinearity of the state transition function $F_{k-1}^{i}(x_{k-1})$ and the measurement function $H_{k}^{i}(x_{k})$, the Gaussian integrations in (22), (23), (37)–(39), (53) and (54) cannot be computed analytically. In this paper, the UT [38] is employed to calculate the Gaussian integrations approximately.

For the Gaussian integrations in (22) and (23), 2n+1 sigma points are generated from the mixing state ${\hat{x}}_{k-1}^{0i}$ and the covariance matrix $P_{k-1}^{0i}$ as

$$\left\{ \begin{array}{l} \chi_{k-1,0}^{0i}={\hat{x}}_{k-1}^{0i}\\ \chi_{k-1,p}^{0i}={\hat{x}}_{k-1}^{0i}+{\left(\sqrt{(n+\lambda )P_{k-1}^{0i}}\right)}_{p}\;p=1,2,\cdots ,n\\ \chi_{k-1,p}^{0i}={\hat{x}}_{k-1}^{0i}-{\left(\sqrt{(n+\lambda )P_{k-1}^{0i}}\right)}_{p}\;p=n+1,n+2,\cdots ,2n \end{array}\right.,$$

where ${\left(\sqrt{P}\right)}_{p}$ denotes the *p*-th column of the matrix square root of P, $\lambda =\alpha^{2}(n+\kappa )-n$ is a scaling parameter, α controls the divergence of the sigma points and is usually set to a small positive value (e.g., 0.01), κ is a secondary scaling parameter which is usually set to zero. Then, the predicted state ${\hat{x}}_{k|k-1}^{i}$ and the covariance matrix $P_{k|k-1}^{i}$ are calculated as

(62) $${\hat{x}}_{k|k-1}^{i}=\sum_{p=0}^{2n}w_{m,p}F_{k-1}^{i}(\chi_{k-1,p}^{0i}),$$

(63) $$P_{k|k-1}^{i}=\sum_{p=0}^{2n}w_{c,p}(F_{k-1}^{i}(\chi_{k-1,p}^{0i})-{\hat{x}}_{k|k-1}^{i}){\left(F_{k-1}^{i}(\chi_{k-1,p}^{0i})-{\hat{x}}_{k|k-1}^{i}\right)}^{T}+Q_{k-1}^{i},$$

where the weights of sigma points are given by

$$\left\{ \begin{array}{l} w_{m,0}=\lambda /(n+\lambda )\\ w_{c,0}=\lambda /(n+\lambda )+(1-\alpha^{2}+\beta )\\ w_{m,p}=w_{c,p}=0.5/(n+\lambda )\;p=1,2,\cdots ,2n \end{array}\right.,$$

where β is used to incorporate the prior information of the distribution. β=2 is optimal under Gaussian distributions.

For the Gaussian integrations in (37)–(39), the sigma points generated from the predicted state ${\hat{x}}_{k|k-1}^{i}$ and the covariance matrix $P_{k|k-1}^{i}$ are given by

$$\left\{ \begin{array}{l} \chi_{k|k-1,0}^{i}={\hat{x}}_{k|k-1}^{i}\\ \chi_{k|k-1,p}^{i}={\hat{x}}_{k|k-1}^{i}+{\left(\sqrt{(n+\lambda )P_{k|k-1}^{i}}\right)}_{p}\;p=1,2,\cdots ,n\\ \chi_{k|k-1,p}^{i}={\hat{x}}_{k|k-1}^{i}-{\left(\sqrt{(n+\lambda )P_{k|k-1}^{i}}\right)}_{p}\;p=n+1,n+2,\cdots ,2n \end{array}\right.$$

Then, the predicted measurement mean ${\hat{z}}_{k|k-1}^{i}$, the covariance matrix $P_{zz,k|k-1}^{i}$ and the cross-covariance matrix $P_{xz,k|k-1}^{i}$ are calculated as

(64) $${\hat{z}}_{k|k-1}^{i}=\sum_{p=0}^{2n}w_{m,p}H_{k}^{i}(\chi_{k|k-1,p}^{i}),$$

(65) $$P_{zz,k|k-1}^{i}=\sum_{p=0}^{2n}w_{c,p}(H_{k}^{i}(\chi_{k|k-1,p}^{i})-{\hat{z}}_{k|k-1}^{i}){\left(H_{k}^{i}(\chi_{k|k-1,p}^{i})-{\hat{z}}_{k|k-1}^{i}\right)}^{T}+{\tilde{R}}_{k},$$

(66) $$P_{xz,k|k-1}^{i}=\sum_{p=0}^{2n}w_{c,p}(\chi_{k|k-1,p}^{i}-{\hat{x}}_{k|k-1}^{i}){\left(H_{k}^{i}(\chi_{k|k-1,p}^{i})-{\hat{z}}_{k|k-1}^{i}\right)}^{T}$$

We generate the sigma points from the posterior state ${\hat{x}}_{k}^{i}$ and the covariance matrix $P_{k}^{i}$ as

$$\left\{ \begin{array}{l} \chi_{k,0}^{i}={\hat{x}}_{k}^{i}\\ \chi_{k,p}^{i}={\hat{x}}_{k}^{i}+{\left(\sqrt{(n+\lambda )P_{k}^{i}}\right)}_{p}\;p=1,2,\cdots ,n\\ \chi_{k,p}^{i}={\hat{x}}_{k}^{i}-{\left(\sqrt{(n+\lambda )P_{k}^{i}}\right)}_{p}\;p=n+1,n+2,\cdots ,2n \end{array}\right..$$

Then, the Gaussian integrations in (53) and (54) are calculated as

(67) $${\hat{z}}_{k}^{i}=\sum_{p=0}^{2n}w_{m,p}H_{k}^{i}(\chi_{k,p}^{i}),$$

(68) $$A_{k}^{i}=(z_{k}-{\hat{z}}_{k}^{i}){\left(z_{k}-{\hat{z}}_{k}^{i}\right)}^{T}+\sum_{p=0}^{2n}w_{c,p}(H_{k}^{i}(\chi_{k,p}^{i})-{\hat{z}}_{k}^{i}){\left(H_{k}^{i}(\chi_{k,p}^{i})-{\hat{z}}_{k}^{i}\right)}^{T}$$

With the above derivations, the proposed robust IMM filter is summarized as follows:

**Step 1:** Choose initial estimates ${\hat{x}}_{0}^{i}$, $P_{0}^{i}$, $u_{0}^{i}$, ${\hat{a}}_{0}^{i}$, ${\hat{b}}_{0}^{i}$, ${\hat{t}}_{0}^{i}$ and $T_{0}^{i}$ for each mode i Set $\pi_{ij}$ and ρ. Let k=1

**Step 2:** Calculate the predicted mode probability $u_{k|k-1}^{j}$ and the mixing mode probability $u_{k-1}^{ij}$ using (4) and (5). Then, calculate the mixing quantities ${\hat{x}}_{k-1}^{0i}$, $P_{k-1}^{0i}$, ${\hat{t}}_{k-1}^{0i}$, $T_{k-1}^{0i}$, ${\hat{a}}_{k-1}^{0i}$ and ${\hat{b}}_{k-1}^{0i}$ for each mode i using (8)–(11), (16) and (17).

**Step 3:** Calculate the predicted quantities ${\hat{x}}_{k|k-1}^{i}$, $P_{k|k-1}^{i}$, ${\hat{t}}_{k|k-1}^{i}$, $T_{k|k-1}^{i}$, ${\hat{a}}_{k|k-1}^{i}$ and ${\hat{b}}_{k|k-1}^{i}$ for each mode i using (62), (63) and (24)–(27).

**Step 4:** Update the posterior quantities ${\hat{x}}_{k}^{i}$, $P_{k}^{i}$, ${\hat{t}}_{k}^{i}$, $T_{k}^{i}$, ${\hat{a}}_{k}^{i}$ and ${\hat{b}}_{k}^{i}$ for each mode i through fixed point iterations based on VB approach as follows:

Update ${\hat{t}}_{k}^{i}$ and ${\hat{a}}_{k}^{i}$ using (43) and (49), and initialize ${\hat{x}}_{k}^{i}={\hat{x}}_{k|k-1}^{i}$, $P_{k}^{i}=P_{k|k-1}^{i}$, $T_{k}^{i}=T_{k|k-1}^{i}$, ${\hat{b}}_{k}^{i}={\hat{b}}_{k|k-1}^{i}$.

Repeat

   Update $A_{k}^{i}$ using (67) and (68).

   Update ${\hat{\alpha }}_{k}^{i}$ and ${\hat{\beta }}_{k}^{i}$ using (46), (47), (52) and (55).

   Update $T_{k}^{i}$ and ${\hat{b}}_{k}^{i}$ using (44), (50), (51) and (56).

   Update ${\hat{x}}_{k}^{i}$ and $P_{k}^{i}$ using (34), (36), (40), (51), (52) and (64)–(66).

Until converged

**Step 5:** Update the posterior mode probability $u_{k}^{i}$ using (57) and (59).

**Step 6:** Calculate ${\hat{x}}_{k}$ and $P_{k}$ using (60) and (61). Let k+1→k, and return to Step2.

## 4. Simulation Example

This section presents a two-dimensional maneuvering target tracking scenario with a period of 200 s to demonstrate the performance of the proposed filter. A maneuvering target moves following two models: constant velocity (CV) model and coordinated turn (CT) model. The modes representing the CV model and CT model at time k are denoted by $r_{k}=1$ and $r_{k}=2$, respectively. The state of the CV model is $x_{k}={\left(x_{k},y_{k},{\dot{x}}_{k},{\dot{y}}_{k}\right)}^{T}$ including the components of the position $\left(x_{k},y_{k}\right)$ and the velocity $\left({\dot{x}}_{k},{\dot{y}}_{k}\right)$. The dynamics of the CV model is given by

$$x_{k}=\left(\begin{array}{cc} I_{2} & \Delta tI_{2}\\ 0_{2} & I_{2} \end{array}\right)x_{k-1}+w_{k-1}^{1},$$

where Δt is the sampling period. The covariance matrix of the process noises $w_{k-1}^{1}$ is given by

$$Q_{k-1}^{1}=q_{x}^{1}\left(\begin{array}{cc} {\left(\Delta t\right)}^{3}I_{2}/3 & {\left(\Delta t\right)}^{2}I_{2}/2\\ {\left(\Delta t\right)}^{2}I_{2}/2 & \Delta tI_{2} \end{array}\right),$$

where $q_{x}^{1}$ is the power spectral density. The state of the CT model is $x_{k}={\left(x_{k},y_{k},{\dot{x}}_{k},{\dot{y}}_{k},\omega_{k}\right)}^{T}$, where $\omega_{k}$ is the turn rate. The dynamics of the CT model is given by

$$x_{k}=\left(\begin{array}{ccccc} 1 & 0 & \sin(\omega_{k-1}\Delta t)/\omega_{k-1} & (\cos(\omega_{k-1}\Delta t)-1)/\omega_{k-1} & 0\\ 0 & 1 & (1-\cos(\omega_{k-1}\Delta t))/\omega_{k-1} & \sin(\omega_{k-1}\Delta t)/\omega_{k-1} & 0\\ 0 & 0 & \cos(\omega_{k-1}\Delta t) & -\sin(\omega_{k-1}\Delta t) & 0\\ 0 & 0 & \sin(\omega_{k-1}\Delta t) & \cos(\omega_{k-1}\Delta t) & 0\\ 0 & 0 & 0 & 0 & 1 \end{array}\right)x_{k-1}+w_{k-1}^{2},$$

and the covariance matrix of the process noises $w_{k-1}^{2}$ is given by

$$Q_{k-1}^{2}=\left(\begin{array}{ccc} q_{x}^{2}{\left(\Delta t\right)}^{3}I_{2}/3 & q_{x}^{2}{\left(\Delta t\right)}^{2}I_{2}/2 & 0\\ q_{x}^{2}{\left(\Delta t\right)}^{2}I_{2}/2 & q_{x}^{2}\Delta tI_{2} & 0\\ 0 & 0 & q_{\omega } \end{array}\right),$$

where $q_{x}^{2}$ and $q_{\omega }$ are the power spectral densities corresponding to $\left(x_{k},y_{k},{\dot{x}}_{k},{\dot{y}}_{k}\right)$ and $\omega_{k}$, respectively.

A sensor located at $\left(x_{s},y_{s}\right)$ collects noisy range and azimuth measurements of the target according to the equation

$$z_{k}=\left(\begin{array}{c} \sqrt{{\left(x_{k}-x_{s}\right)}^{2}+{\left(y_{k}-y_{s}\right)}^{2}}\\ \arctan((y_{k}-y_{s})/(x_{k}-x_{s})) \end{array}\right)+\varepsilon_{k}.$$

**Figure 1:**
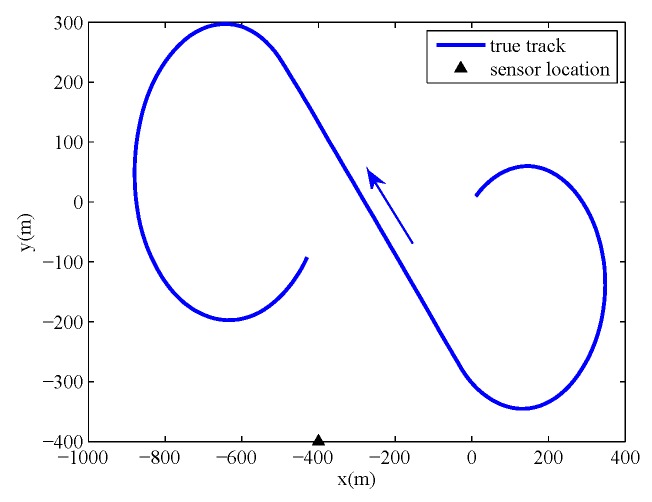
True track of target and sensor location.

The sampling period is Δt=1s. The target executes a −4°/s coordinated turn from 0 s to 70 s, moves at a constant velocity from 71 s to 120 s, and executes a 4°/s coordinated turn from 121 s to 200 s. The power spectral densities are set to $q_{x}^{1}=q_{x}^{2}=0.01\;m^{2}s^{-3}$ and $q_{\omega }=2\times 10^{-6}\;{\mathrm{rad}}^{2}s^{-3}$. The true initial state of target is $x_{0}={\left(0\;m,\;0\;m,\;10\;m/s,\;10\;m/s,\;-4{}^{\circ}/s\right)}^{T}$, and the location of the sensor is $\left(x_{s},y_{s})=(-400\;m,\;-400\;m\right)$. The true track of the target and the sensor location are shown in Figure 1.

To illustrate the performance of the proposed filter, we consider the following four cases under different conditions of measurement noises:

Case A: Gaussian noises

$$\varepsilon_{k}∼N(\varepsilon_{k};0,\bar{R}),$$

where $\bar{R}=\mathrm{diag}({(10m)}^{2},{\left(0.2{}^{\circ}\right)}^{2})$ is the known nominal covariance matrix.

Case B: Gaussian noises with time varying covariance matrix

$$\varepsilon_{k}∼N(\varepsilon_{k};0,(0.1+0.05\cos(2\pi k/100))\bar{R}).$$

This case is used to simulate the situation that the actual covariance matrix is deviated from the nominal one $\bar{R}$.

Case C: Contaminated Gaussian noises

$$\varepsilon_{k}∼(1-\delta )N(\varepsilon_{k};0,\bar{R})+\delta N(\varepsilon_{k};0,100\bar{R}),$$

where $N(\varepsilon_{k};0,\bar{R})$ can be seen as a normal distribution, $N(\varepsilon_{k};0,100\bar{R})$ can be considered as a perturbing distribution with much larger covariance matrix due to outliers, and δ∈[0,1] is a perturbing parameter that represents the extent of the perturbation. This case is used to simulate the corrupted measurements by outliers. We set δ=0.1.

Case D: Contaminated Gaussian noises with time varying covariance matrix

$$\varepsilon_{k}∼(1-\delta )N(\varepsilon_{k};0,(0.1+0.05\cos(2\pi k/100))\bar{R})+\delta N(\varepsilon_{k};0,100\bar{R}).$$

Both outliers and covariance mistuning are simultaneously simulated in this case. δ is also set to 0.1.

Four existing filters including VB based Student’s *t* filter (VBStdF) [30] utilizing only single CT model, IMM filter (IMMF) [1], VB based IMM filter (IMMVBF) estimating the unknown covariance matrix of measurement noises [33], and VB based IMM filter modeling measurement noises as Student’s *t*-distribution (IMMVBStdF) [31] are compared with the proposed filter. 1000 Monte Carlo (MC) runs are carried out for each case of measurement noises above. All the filters are implemented in MATLAB on an Intel i7 3.6GHz processor.

The initial estimates of the degree and the inverse scale matrix with respect to the inverse Wishart distribution in VBStdF, IMMVBF and the proposed filter are chosen as ${\hat{t}}_{0}=7$ and $T_{0}=({\hat{t}}_{0}-m-1)\bar{R}$. All the initial estimates of the Gamma distribution parameters in VBStdF, IMMVBStdF and the proposed filter are set to 0.5. For all the VB approach based filters, ρ=1−exp(−4) is used, and the VB iteration is terminated when the difference of position estimates between two adjacent iterations is less than 1e-6. The mode probabilities are initialized as $u_{0}^{1}=0.1$ and $u_{0}^{2}=0.9$, and the mode transition probability is set to $\pi_{ij}=\left(\begin{array}{ll} 0.99 & 0.01\\ 0.01 & 0.99 \end{array}\right)$ for all the IMM type filters. The initial covariance matrix of the state estimate is set to $P_{0}=\mathrm{diag}(100\;m^{2},\;100\;m^{2},\;1\;{(m/s)}^{2},\;1\;{(m/s)}^{2},\;1\times 10^{-5}\;{\left(\mathrm{rad}/s\right)}^{2})$, and the initial estimate of the state ${\hat{x}}_{0}$ is chosen randomly from the Gaussian distribution $N(\cdot ;x_{0},P_{0})$ for all the filters.

The root mean square errors (RMSEs) and average RMSEs (ARMSEs) of position, velocity and turn rate are used to evaluate the estimation accuracy of the filters. The RMSE and ARMSE of position are defined as

$$\mathrm{RMS}E_{\mathrm{pos}}(k)=\sqrt{\frac{1}{P}\sum_{i=1}^{P}\left({\left({\hat{x}}_{k}^{\left(i\right)}-x_{k}^{\left(i\right)}\right)}^{2}+{\left({\hat{y}}_{k}^{\left(i\right)}-y_{k}^{\left(i\right)}\right)}^{2}\right)},$$

$$\mathrm{ARMS}E_{\mathrm{pos}}=\frac{1}{T}\sum_{k=1}^{T}\mathrm{RMS}E_{\mathrm{pos}}(k).$$

where P is the total count of MC runs, $\left(x_{k}^{\left(i\right)},y_{k}^{\left(i\right)}\right)$ and $\left({\hat{x}}_{k}^{\left(i\right)},{\hat{y}}_{k}^{\left(i\right)}\right)$ are true position and estimated position for the *i*-th MC run, and T is the total count of samples. The RMSEs and ARMSEs of velocity and turn rate are defined similar.

**Figure 2:**
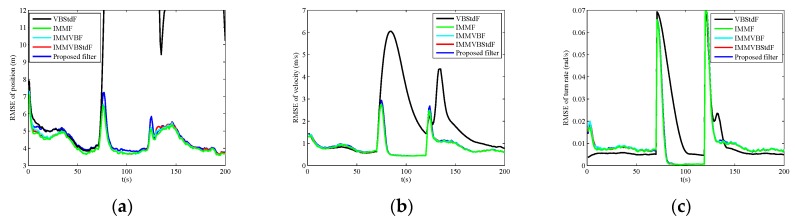
Root mean square errors (RMSEs) versus time in Case A. (**a**) Position; (**b**) Velocity; (**c**) Turn rate.

**Figure 3:**
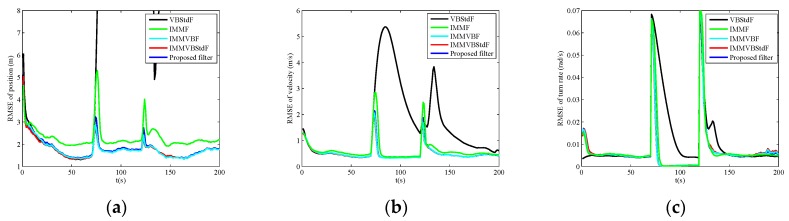
RMSEs versus time in Case B. (**a**) Position; (**b**) Velocity; (**c**) Turn rate.

**Figure 4:**
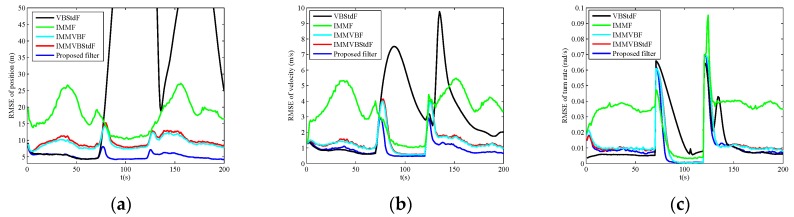
RMSEs versus time in Case C. (**a**) Position; (**b**) Velocity; (**c**) Turn rate.

**Figure 5:**
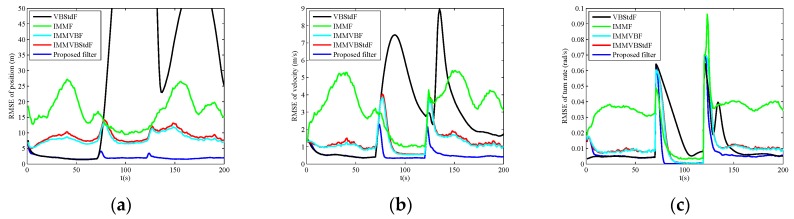
RMSEs versus time in Case D. (**a**) Position; (**b**) Velocity; (**c**) Turn rate.

The RMSEs of position, velocity and turn rate in four cases are shown in Figure 2, Figure 3, Figure 4 and Figure 5, and the corresponding ARMSEs are listed in Table 1. The overall performance of VBStdF in estimation accuracy is the worst among the filters for all cases. We do not plot the entire RMSE curves of position for VBStdF to show other curves distinctly. The largest RMSE of position for VBStdF under four cases are 53.03 m, 50.19 m, 99.82 m and 98.30 m, respectively. The reason for the poor performance of VBStdF is that the single-model based filter is less adaptive to target maneuvering compared with multiple-model based filter.

In Case A, the estimation accuracy of the proposed filter is lower than IMMF and IMMVBF. The reason is that the assumed Student’s *t*-distribution for measurement noises deviates from the actual Gaussian distribution. The performance of the proposed filter in estimation accuracy is also poorer than IMMVBStdF. That is because the scale matrix of Student’s *t*-distribution is required to be estimated additionally for the proposed filter, while the scale matrix is known exactly for IMMVBStdF. However, the accuracy degradation of the proposed filter is not obvious compared with IMMF, IMMVBF, and IMMVBStdF.

In Case B, IMMF has larger RMSEs than IMMVBF, IMMVBStdF, and the proposed filter since IMMF utilizes mistuned covariance matrix of measurement noises, while the IMMVBF and the proposed filters can learn the covariance matrix adaptively. The IMMVBStdF seems to have the ability of capturing the unknown covariance matrix either. The performance of the proposed filter in estimation accuracy is also slightly worse than IMMVBF and IMMVBStdF as Case A.

**Figure 6:**
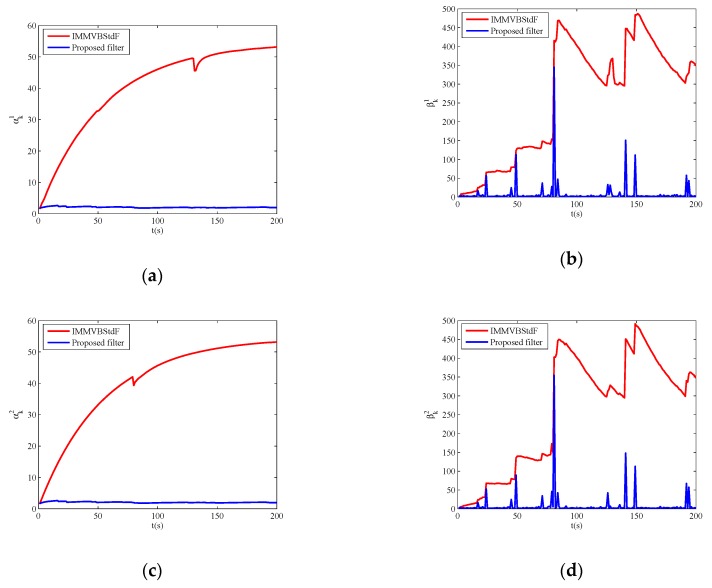
Estimates of degrees of freedom parameters versus time. (**a**) ${\hat{\alpha }}_{k}^{1}$; (**b**) ${\hat{\beta }}_{k}^{1}$; (**c**) ${\hat{\alpha }}_{k}^{2}$; (**d**) ${\hat{\beta }}_{k}^{2}$.

In Case C, the proposed filter outperforms other filters obviously in estimation accuracy. IMMF and IMMVBF are based on the assumption of Gaussian noises so that they cannot cope with heavy-tailed measurement noises. Figure 6 shows the estimates of the dof parameters ${\hat{\alpha }}_{k}^{1}$, ${\hat{\beta }}_{k}^{1}$, ${\hat{\alpha }}_{k}^{2}$ and ${\hat{\beta }}_{k}^{2}$ versus time for IMMVBStdF and the proposed filter in one MC run. We can see that the estimates of dof parameters for IMMVBStdF become large after several time steps. Therefore, the distribution of measurement noises converges to a Gaussian distribution and loses the heavy-tailed property. The estimates of dof parameters for the proposed filter maintain small values overall, except some high peaks for ${\hat{\beta }}_{k}^{1}$ and ${\hat{\beta }}_{k}^{2}$ curves caused by outliers. Therefore, the proposed filter is more robust against heavy-tailed measurement noises than IMMVBStdF. In addition, we can see that the IMMVBF and IMMVBStdF are more robust than IMMF.

The results under Case D are similar to Case C.

**Figure 7:**
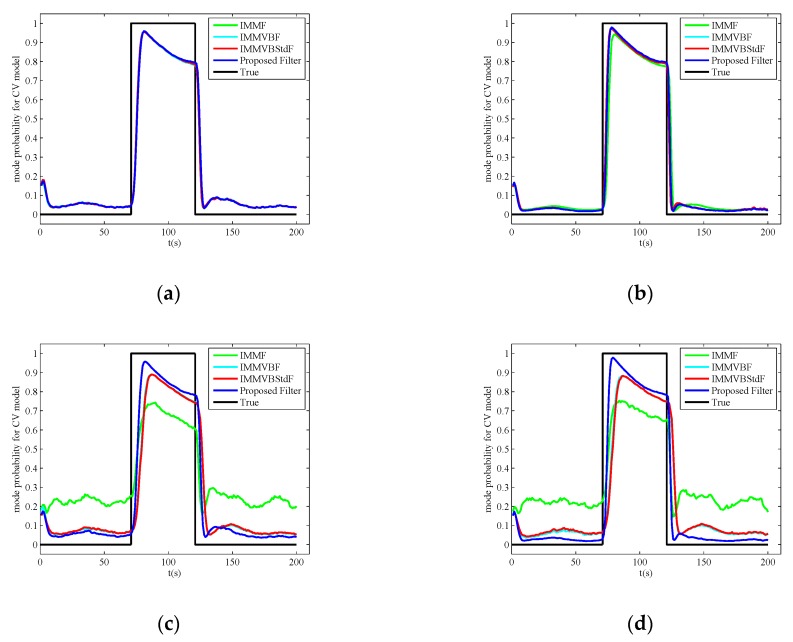
1000 Monte Carlo runs averaged estimates of mode probabilities for constant velocity model in four cases. (**a**) Case A; (**b**) Case B; (**c**) Case C; (**d**) Case D.

The 1000 MC runs averaged estimates of mode probabilities for CV model are shown in Figure 7. In Case A, we can see that the estimation results are nearly the same for all the filters. In Case B, the accuracy of IMMF estimates is lower than other filters and the response to mode changes for IMMF is more lagging than other filters. The reason is also that the covariance matrix of the measurement noises utilized by IMMF is mistuned. In Case C and D, the proposed filter achieves more accuracy estimates of mode probabilities than other filters, indicating that the proposed filter can also improve the estimation accuracy of mode probability under heavy-tailed measurement noises.

We use the number of floating points operations (flops) [39] to measure the computational complexity of filter. The computational complexity of VBStdF, IMMF, IMMVBF, IMMVBStdF and the proposed filter are listed in Table 2. We can see that the proposed filter has the highest computational complexity among the filters. According to Table 2, the computational complexity of proposed filter is higher than VBStdF mainly because of multiple model operations, and is higher than IMMF mainly because of fixed point iterations. The proposed filter requires more flops compared with IMMVBF and IMMVBStdF since the proposed filter involves more parameters to be estimated in the filter recursions.

We further test the computational cost of filters for the above tracking scenario. The 1000 MC runs averaged computation time in four cases are shown in Table 3. It can be seen that the proposed filter expends more computational cost than other filters, demonstrating that the proposed filter has higher computational complexity than other filters.

## 5. Conclusions

We propose a robust IMM filter against heavy-tailed measurement noises for maneuvering target tracking. The heavy-tailed measurement noises are treated as Student’s *t*-distribution, and the unknown dof and scale matrix are assumed to be governed by Gamma and inverse Wishart distributions, respectively. Then, the filter recursions for the target state and the parameters of Gamma and inverse Wishart distributions are designed in the IMM framework. In the model interaction step, the mixing distributions of the state, dof, and scale matrix are achieved by using the methods of matching the first two moments and minimizing weighted KL divergence. In the measurement update step, the state and the unknown parameters are jointly estimated by employing the VB approach. The problem of system nonlinearity is solved by utilizing UT to compute the Gaussian integrations approximately. Simulation results show that the proposed filter outperforms other related filters in terms of the estimation accuracy for the state and the mode probability under heavy-tailed measurement noises.

The main contributions of this paper are summarized as follows. Firstly, a robust IMM filter is proposed to properly handle both model uncertainty and outlier measurements. Secondly, by treating the unknown dof of Student’s *t*-distribution of heavy-tailed measurement noises as a Gamma distributed random variable, the heavy-tailed property of Student’s *t*-distribution is maintained and the robustness is improved over the existing filter. Finally, the problem of system nonlinearity is addressed by using UT technology.

## Figures and Tables

**Table 1:** Average root mean square errors of filters in four cases.

	Case	VBStdF	IMMF	IMMVBF	IMMVBStdF	Proposed Filter
**Position (m)**	A	19.9747	4.3292	4.3755	4.3812	4.5475
	B	17.1240	2.3064	1.7662	1.7485	1.8055
	C	39.8238	17.5279	9.1497	9.7692	5.1772
	D	37.3586	16.9337	8.1700	8.9868	2.0456
**Velocity (m/s)**	A	1.8595	0.8287	0.8410	0.8361	0.8627
	B	1.5341	0.6204	0.5097	0.5018	0.5136
	C	2.9977	3.3905	1.3687	1.4143	0.9405
	D	2.7279	3.3061	1.2562	1.3375	0.5493
**Turn rate (rad/s)**	A	0.0125	0.0098	0.0100	0.0099	0.0101
	B	0.0111	0.0077	0.0071	0.0072	0.0071
	C	0.0149	0.0316	0.0130	0.0130	0.0106
	D	0.0138	0.0309	0.0122	0.0125	0.0073

**Table 2:** Computational complexity of filters, where *m* denotes the dimension of measurements, *n* denotes the dimension of target state, *M* denotes the number of models for interacting multiple model type filters, and *N* denotes the number of fixed point iterations for variational Bayesian approach based filters.

Filter	Number of Flops
**VBStdF**	(4*m*^3^ + 2*n*^3^/3 + 14*m*^2^*n* + 20*mn*^2^ + 10*m*^2^ + 8*n*^2^ + 15*mn* + 4*m* + *n* + 15)*N* + 31*n*^3^/3 + *m*^2^ + 12*n*^2^ + 5*n*
**IMMF**	(4*n*^2^ + 3*n* + 5)*M*^2^ + (4*m*^3^/3 + 32*n*^3^/3 + 8*m*^2^*n* + 12*mn*^2^ + 5*m*^2^ + 19*n*^2^ + 9*mn* + 4*m* + 4*n* + 6)*M* + 3*n*
**IMMVBF**	(*m*^3^ + 2*n*^3^/3 + 14*m*^2^*n* + 20*mn*^2^ + 9*m*^2^ + 8*n*^2^ + 15*mn* + 3*m* + *n* + 3)*MN* + (2*m*^2^ + 4*n*^2^ + 3*n* + 7)*M*^2^ + (8*m*^3^/3 + 14*n*^3^ + 15*n*^2^ + 3*m* + 7*n* + 33)*M* + 3*n*
**IMMVBStdF**	(3*m*^3^ + 2*n*^3^/3 + 14*m*^2^*n* + 20*mn*^2^ + 6*m*^2^ + 8*n*^2^ + 15*mn* + 4*m* + *n* + 1)*MN* + (4*n*^2^ + 3*n* + 13)*M*^2^ + (14*n*^3^ + 15*n*^2^ + 7*n* + 39)*M* + 3*n*
**Proposed filter**	(4*m*^3^ + 2*n*^3^/3 + 14*m*^2^*n* + 20*mn*^2^ + 10*m*^2^ + 8*n*^2^ + 15*mn* + 4*m* + *n* + 15)*MN* + (2*m*^2^ + 4*n*^2^ + 3*n* + 15)*M*^2^ + (8*m*^3^/3 + 14*n*^3^ + 15*n*^2^ + 3*m* + 7*n* + 68)*M* + 3*n*

**Table 3:** Computation time of filters in four cases.

Case	VBStdF	IMMF	IMMVBF	IMMVBStdF	Proposed Filter
**A**	0.3900 s	0.1092 s	0.5304 s	0.4992 s	0.8736 s
**B**	0.3588 s	0.1092 s	0.4836 s	0.4836 s	0.8424 s
**C**	0.5148 s	0.1092 s	0.4992 s	0.4524 s	1.1076 s
**D**	0.4836 s	0.1092 s	0.4524 s	0.4368 s	0.9516 s
